# Acute phase response in two consecutive experimentally induced *E. coli *intramammary infections in dairy cows

**DOI:** 10.1186/1751-0147-50-18

**Published:** 2008-06-13

**Authors:** Leena Suojala, Toomas Orro, Hanna Järvinen, Johanna Saatsi, Satu Pyörälä

**Affiliations:** 1 Department of Production Animal Medicine, Faculty of Veterinary Medicine, University of Helsinki, FI-04920 Saarentaus, Finland; 2 Department of Animal Health and Environment, Estonian University of Life Sciences, Kreutzwaldi 62, EE-51014 Tartu, Estonia

## Abstract

**Background:**

Acute phase proteins haptoglobin (Hp), serum amyloid A (SAA) and lipopolysaccharide binding protein (LBP) have suggested to be suitable inflammatory markers for bovine mastitis. The aim of the study was to investigate acute phase markers along with clinical parameters in two consecutive intramammary challenges with *Escherichia coli *and to evaluate the possible carry-over effect when same animals are used in an experimental model.

**Methods:**

Mastitis was induced with a dose of 1500 cfu of *E. coli *in one quarter of six cows and inoculation repeated in another quarter after an interval of 14 days. Concentrations of acute phase proteins haptoglobin (Hp), serum amyloid A (SAA) and lipopolysaccharide binding protein (LBP) were determined in serum and milk.

**Results:**

In both challenges all cows became infected and developed clinical mastitis within 12 hours of inoculation. Clinical disease and acute phase response was generally milder in the second challenge. Concentrations of SAA in milk started to increase 12 hours after inoculation and peaked at 60 hours after the first challenge and at 44 hours after the second challenge. Concentrations of SAA in serum increased more slowly and peaked at the same times as in milk; concentrations in serum were about one third of those in milk. Hp started to increase in milk similarly and peaked at 36–44 hours. In serum, the concentration of Hp peaked at 60–68 hours and was twice as high as in milk. LBP concentrations in milk and serum started to increase after 12 hours and peaked at 36 hours, being higher in milk. The concentrations of acute phase proteins in serum and milk in the *E. coli *infection model were much higher than those recorded in experiments using Gram-positive pathogens, indicating the severe inflammation induced by *E. coli*.

**Conclusion:**

Acute phase proteins would be useful parameters as mastitis indicators and to assess the severity of mastitis. If repeated experimental intramammary induction of the same animals with *E. coli *is used in cross-over studies, the interval between challenges should be longer than 2 weeks, due to the carry-over effect from the first infection.

## Background

Environmental mastitis caused by coliform bacteria is an increasing problem for the dairy industry in many countries [1,2]. Mastitis caused by *Escherichia coli *is typically self-limiting and of short duration, but can be associated with severe clinical signs, reductions in milk yield and heavy tissue damage to mammary gland [3-5]. The strategies for preventing coliform mastitis include hygiene measures and in some countries prophylactic immunization. Incidence and severity of clinical signs of coliform mastitis were reduced using *Escherichia coli *core antigen vaccine [6-8].

Bacterial lipopolysaccharide (LPS), from the cell wall of Gram-negative bacteria, is considered to cause most pathophysiological reactions during coliform mastitis. In coliform mastitis, the severity of clinical signs is considered to depend mainly on the host response [3]. LPS triggers formation of proinflammatory cytokines, produced predominantly by monocytes and macrophages [9,10]. Cytokines initiate the inflammatory response, which induces the acute phase response (APR) by activating the production of acute phase proteins (APP) such as serum amyloid-A (SAA), haptoglobin (Hp) and LPS-binding protein (LBP) [11-15].

Concentrations of two major bovine APP, Hp and SAA, were shown to increase in serum [16,13,14,18] as in milk during mastitis [11,13,19,20]. Hp is mostly secreted by liver cells, but also local production has been demonstrated [15,21]. The other major APP of the cow, SAA, is synthesized by the liver, but also locally by the mammary gland [22-24]. Hp and SAA have been suggested to be suitable inflammatory markers for bovine mastitis [25,26]. LBP is a relatively new inflammatory indicator for mastitis [12].

The aim of this study was to investigate APR in an experimental *E. coli *mastitis model with mastitis induced twice at an interval of two weeks and to evaluate the possible carry-over effect when the same animals are used. Several APP were monitored in serum and milk to study the host response to the bacterial challenge.

## Methods

### Animals and experimental design

Seven clinically healthy lactating (on average 92 days from parturition, range 30–123 days) primiparous cows (three Finnish Ayrshire and four Holstein-Friesian) were used as experimental animals. Experimental *Escherichia coli *mastitis was induced in one quarter of each cow twice at an interval of 14 days. The cows were housed in tie stalls and accustomed to the environment and handling for two weeks before the experiment. The cows were fed with good quality hay, silage and concentrated grain according to their energy requirements. Water was available *ad libitum*. The cows were milked twice a day, at 8 am and 4 pm. The milk composite somatic cell count (SCC) of the cows was less than 100 000 cells/ml and no bacteria were isolated from the milk before the challenges. Mean SCC in the milk of the test quarter before the first challenge was 15 200 cells/ml (range 3 000–57 000 cells/ml) and before the second challenge 14 300 cells/ml (range 5 000–25 000 cells/ml), respectively. Milk yield of the inoculated quarter before the first challenge was on average 3.8 kg (range 3–5.1 kg) and before the second 3.8 kg (range 3–5.3 kg). Mean total daily milk yield was 24.2 kg before the first challenge (range 18.6–31.5 kg) and 22.9 kg before the second challenge (range 15.7–33.0 kg). All cows were treated with flunixin meglumine (dose 2.2 mg/kg) once at 12 hours post challenge (PC), when the first clinical signs were observed, to comply with animal welfare requirements. The Ethics Committee of the Faculty of Veterinary Medicine, Helsinki, Finland approved the study protocol.

The *Escherichia coli *strain, FT238, isolated from clinical mastitis and used previously, was selected for experimental inductions [27,28]. The inoculation dose was prepared as described before [29,28]. One udder quarter of each cow was infused via the teat canal with an average dose of 1500 cfu of *E. coli *(range 1400–1600 cfu) and the inoculation was repeated after 14 days in another udder quarter. The quarters were infused four hours after the evening milking.

### Blood and milk samples

Blood samples were collected from the jugular vein of each cow before challenge and 12, 16, 20, 24, 36, 44, 60, 68 and 156 hours post challenge (PC). Serum was separated and kept frozen at -70°C for later determinations of SAA, Hp and LBP. EDTA blood was collected for leukocyte count (WBC) determination. Aseptic milk samples were collected from the experimental and contralateral quarter before the challenge and 12, 20, 36, 44, 60, 68, 84,108, 132 and 156 hours PC for bacteriology, SCC, N-asetyl-β-D-glucosaminidase (NAGase) activity, SAA, Hp and LBP determinations.

### Clinical observations

Systemic and local signs were monitored throughout the experimental period of 6 days: during the first day every 4 hours and thereafter twice a day at the time of milking. Heart rate, rectal temperature, rumen motility, appetite and general attitude were evaluated. The systemic signs were scored on a three point scale, 1 = no signs to 3 = severe signs; half values were also used [26]. The udder was palpated for soreness, swelling, hardness and temperature, and appearance of milk assessed visually for clots, colour changes and changes in consistency. The local signs were scored on the same scale as systemic signs: milk 1 = normal to 3 = serous or clotty milk and udder 1 = no changes to 3 = severe swelling and soreness in the quarter. Cows with scores ≤1.5 were recorded as having mild mastitis, those with scores >1.5 but ≤2.5 as having moderate mastitis and those with scores from >2.5 to 3 as having severe mastitis. The milk yield from the experimental quarter and the total milk yield were measured before challenge and thereafter until the end of the experimental period.

### Analytical methods for indicators of inflammation

Bacterial counts in the milk were determined by preparation of 10-fold dilution series of milk in sterile saline. Bacteria were cultured on blood agar at 37°C for 24 hours using serial dilutions and counted using a routine plate count method. Milk SCC was measured in Valio Ltd. Laboratories, Finland using a fluoro-optical method (Fossomatic-instrument, Foss Electric, Hillerød, Denmark). SCC values over 30 × 10^6 ^cells/ml were recorded as 30 × 10^6 ^cells/ml. Milk NAGase activity was measured using the fluorogenic method of Kitchen and co-workers (1978) [30] with a microplate modification developed by Mattila [31]. Inter-assay and intra-assay CVs for NAGase activity were for the high control <5% and for the low control 7%. Values over 2.5 pmol/min/ml were expressed as >2.5 pmol/min/ml.

The concentration of SAA in serum and milk was determined by using a commercial ELISA test (Tridelta Development, Wicklow, Ireland). Serum and milk samples were initially diluted 1:500 and 1:50, respectively. For very high SAA values, samples were diluted as necessary up to 1:5000 for serum samples and up to 1:15000 for milk (maximum concentrations 750 mg/l and 2250 mg/l, respectively). The inter-assay and intra-assay coefficients of variation (CV) for SAA analysis were <10% and <7%. Milk and serum Hp concentrations were determined using the method based on the ability of Hp to bind to haemoglobin [32] and using tetramethylbenzidine as the substrate [33]. The assay is aimed for determining of Hp in serum but was here adapted to be used for milk [34]. Optical densities of the formed complex were measured using a spectrophotometer at 450 nm (Multiskan MS, Labsystems, Vantaa, Finland). Lyophilized bovine acute phase serum was used as a standard and calibration was according to the European Union concerted action on standardization of animal APPs (number QLK5-CT-1999-0153). The inter-assay and intra-assay CVs for Hp analysis were <10% and <12%.

LBP concentrations in serum and milk were determined with a commercially available LBP ELISA kit, cross-reacting with bovine LBP (LBP ELISA for various species, Hycult Biotechnology, Uden, The Netherlands). Milk and serum samples were initially diluted 1:500 and 1:1000 respectively, and assayed following the instructions of the manufacturer. For high concentrations, milk was diluted up to 1:5000 and serum up to 1:2000. The optical density at 450 nm and a correction wavelength of 550 nm were measured on a spectrophotometer (Multiskan MS, Labsystems). The LBP concentration was determined by extrapolation using linear regression from a standard curve of known human LBP concentrations. The inter-assay and intra-assay CVs for LBP analysis were <13% and <9%.

Leukocyte count (WBC) was determined 24 hours after sampling using an automated multiparameter analyzer with software for animal samples (Cell-Dyn 3700 System, Abbot Diagnostic Division, Abbot Park, IL, USA).

### Statistical analysis

Linear random-intercept models were used to explore time trend differences between challenge times in milk production data, milk SCC, milk NAGase, WBC and all APP measurements. Bacterial counts in milk and local and systemic sign differences between challenges were tested using generalized linear mixed models in which a Poisson distribution was used for response variables. The cow was included as a random factor. Polynomials for time in increasing order and their interactions with challenge occasion were fixed factors and were added until significant, for modeling changes in time at both challenges. Overall time trend differences between challenges were tested with an F-test. As there were different intervals between sampling, isotropic spatial exponential correlation structures were used for modeling serial correlations of repeated measurements within cows. Logarithmic transformation of milk SCC, NAGase and APPs in milk and serum was used. The nlme-package [35] with statistical software R 2.5.0 [36] was used for fitting linear random-intercept models and generalized linear mixed models were fitted using the GLIMMIX procedure [37] software with the SAS/STAT 9.1 (SAS Institute Inc., Cary, NC, USA).

## Results

### Clinical findings

**Figure 1:**
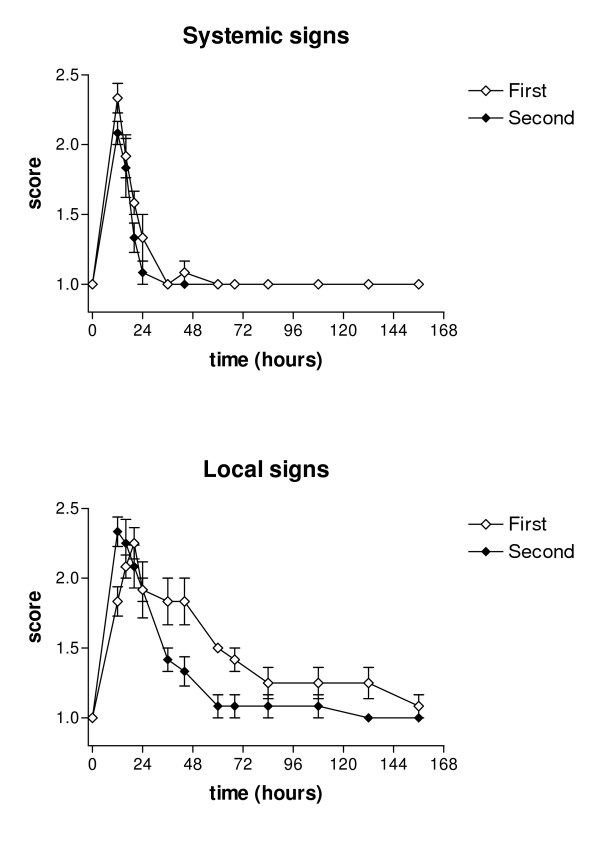
**Mean scores for systemic and local clinical signs in two consecutive *E. coli *challenges**. Systemic and local clinical signs following two consecutive intramammary challenges with *E. coli *at an interval of two weeks. Values are mean scores for six cows with SEM represented by vertical bars.

After both challenges all cows became infected and developed clinical mastitis within 12 hours after inoculation. One cow was excluded from the experiment because of acute spontaneous coliform mastitis after the first challenge. All cows showed systemic and local inflammatory response after both challenges. Systemic response began within 12 hours, being moderate in all cows at 12 hours PC based on the clinical severity scoring system. Systemic signs disappeared in cows after both challenges until 36 hours PC. Local signs were still recorded at the end of the experimental period of 6 d after the first challenge, but disappeared by 60 hours PC after the second challenge. In both challenges, cows developed a similar systemic response, but their local responses varied more. After the second challenge, local clinical signs were significantly milder (*p *< 0.05) but no statistically significant differences were noted in systemic signs (Figure 1).

### Milk production

**Figure 2:**
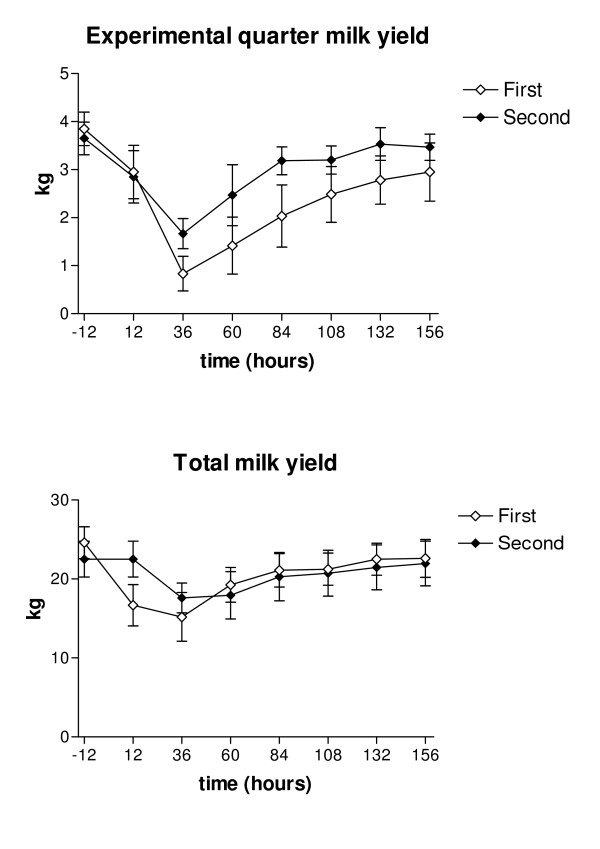
**Mean total daily milk yield and milk yield of the experimentally infected quarter in two consecutive *E. coli *challenges**. Total daily milk yield (kg) and milk yield (kg) of the experimentally infected quarter following two consecutive intramammary challenges with *E. coli *at an interval of two weeks. Values are means for six cows with SEM represented by vertical bars.

The daily milk yield was at its lowest 36 hours PC after both challenges, being on average 16 kg after the first challenge and 17.1 kg after the second. After 6 days PC the total milk yields in both groups returned to pre-challenged levels. The total daily milk yield during the experimental period was significantly higher for the second challenge (*p *< 0.05). The milk yield of the infected quarter was lowest at 36 hours PC, being 1.1 kg (range 0 – 2.5 kg) after the first challenge and 1.4 kg (range 0.8 – 2.5 kg) after the second. The milk yield from infected quarters was significantly higher after the second challenge (*p *< 0.05; Figure 2).

### Bacterial counts in milk

**Figure 3:**
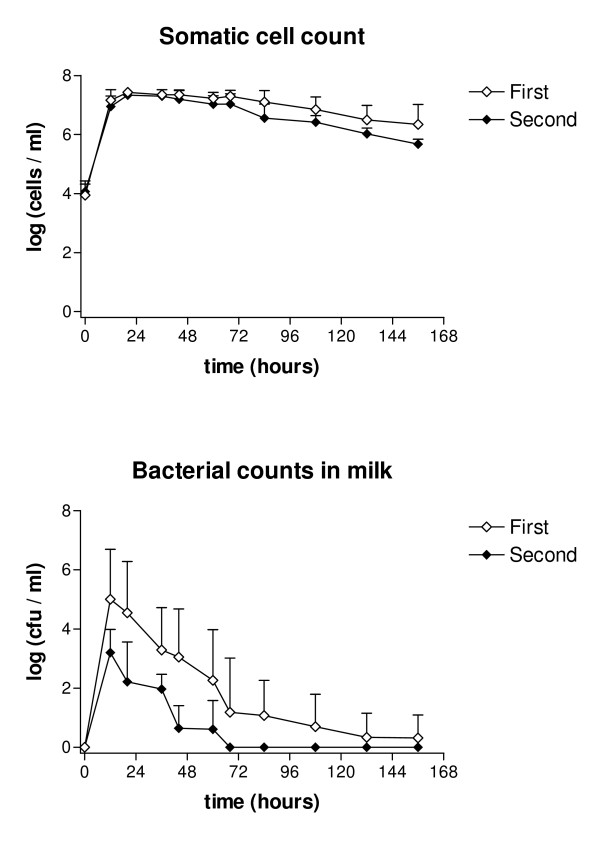
**Mean somatic cell counts and bacterial counts in milk in two consecutive *E. coli *challenges**. Mean somatic cell counts (log cells/ml) and bacterial counts (log cfu/ml) in milk following two consecutive intramammary challenges with *E. coli *at an interval of two weeks. Values are means for six cows with SEM represented by vertical bars.

Bacterial counts in the milk of the challenged quarters peaked at 12 hours PC at both challenge times, being on average 18.1 × 10^6 ^cfu/ml in the first challenge and 6800 cfu/ml in the second challenge. Bacteria were still isolated in low numbers from one cow (80 cfu/ml) 6 days PC after the first challenge, but after the second challenge were eliminated totally in all cows within 68 hours. Overall bacterial counts were lower at the second challenge (*p *< 0.05; Figure 3).

### Indicators of inflammation in the milk

Milk SCC of the challenged quarters started to increase from the baseline values after both challenges within 12 hours and reached the maximum level at 20 hours PC, being over 25 × 10^6 ^cells/ml after first challenge and 20.7 × 10^6 ^cells/ml after the second. In both groups SCC gradually decreased after challenges. At the end of the experimental period of 6 d, SCC was on average 5.8 × 10^6 ^cells/ml (range 541 000 – 18.1 × 10^6 ^cells/ml) after the first challenge and 541 000 cells/ml (range 256 000–705 000 cells/ml) after the second challenge. The difference between the groups was not statistically significant (Figure 3).

**Figure 4:**
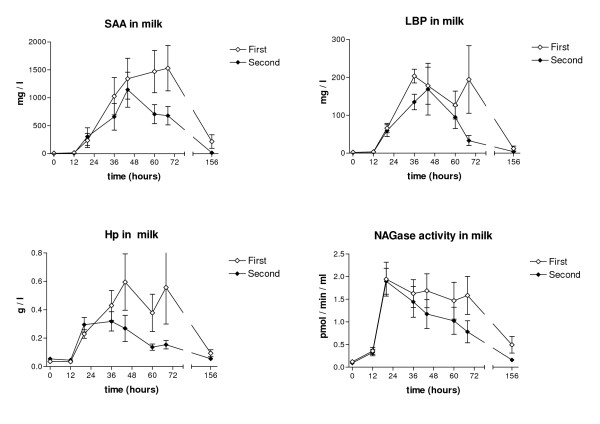
**Concentrations of SAA, LBP, Hp and NAGase activity in milk in two consecutive *E. coli *challenges**. Mean concentrations of SAA, LBP, Hp and NAGase activity in milk following two consecutive intramammary challenges with *E. coli *at an interval of two weeks. Values are means for six cows with SEM represented by vertical bars.

NAGase activity of the milk after both challenges peaked at 20 hours PC, being on average 1.95 pmol/min/μl (range 0.65 – >2.5) after the first challenge and 1.90 pmol/min/μl after the second (range 0.63 – >2.5). After the first challenge NAGase activity remained elevated over the experimental period, but returned to the baseline value by this time after the second challenge. The difference between the challenges was not statistically significant (Figure 4). Milk SCC and NAGase activity in the contralateral control quarters remained at the pre-challenged levels in both groups after both challenges.

Before the first challenge, mean milk SAA concentrations were 7.1 mg/l ± 11.0 mg/l and before the second, 0.4 mg/l ± 0.4 mg/l. Milk SAA concentrations in both groups started to increase after 12 hours PC and reached the maximum (mean 1315.9 mg/l ± 947.3) at 60 hours PC after the first challenge and at 44 hours PC (mean 925.0 mg/l ± 609.1) after the second challenge. After the second challenge, SAA concentration decreased faster: mean concentration by the end of the experimental period was 16.6 ± 11.9 mg/l. Milk Hp started to increase after both challenges 12 hours PC and peaked at 44 hours at 0.60 g/l (± 0.49 g/l) after the first challenge and at 36 hours at 0.32 g/l (± 0.17 g/l) after second challenge. The Hp concentrations in milk returned to background levels within 156 hours after both challenges, faster after the second challenge. LBP concentrations in milk started to rise 12 hours PC and peaked at 36 hours PC, being on average 203.5 ± 44.3 mg/l after the first challenge and 169.0 ± 167.7 mg/l after the second. LBP was still increasing 6 d after the first challenge, but had reached the pre-challenge level by that time after the second challenge. Statistically significant differences between the two challenges were established for milk SAA (*p *< 0.05) and Hp (*p *< 0.05).

### Indicators of inflammation in blood

The concentrations of SAA in serum started to rise slowly after challenges until 24 hours PC, concentrations peaking after the first challenge by 60 hours PC (mean 447.9 mg/l ± 164.8) and after the second challenge by 44 hours PC (mean 307.1 mg/l ± 66.2). In both groups the SAA in serum subsequently decreased gradually, but had not reached the base levels by the end of the experimental period. However, there were no statistically significant differences between the two challenges.

**Figure 5:**
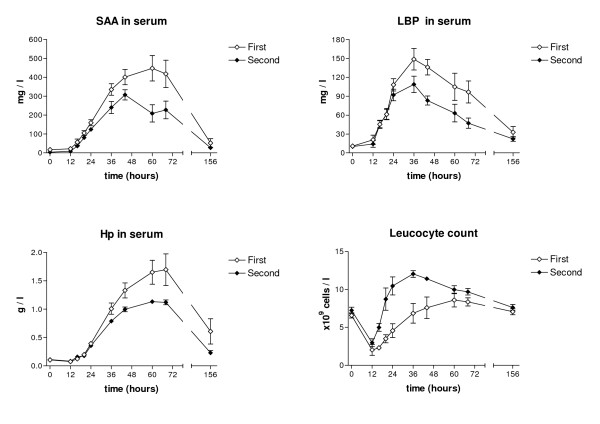
**Concentrations of SAA, LBP and Hp in serum and blood leukocyte counts in two consecutive *E. coli *challenges**. Mean concentrations of SAA, LBP and Hp in serum and mean blood leukocyte counts following two consecutive intramammary challenges with *E. coli *at an interval of two weeks. Values are means for six cows with SEM represented by vertical bars.

The same trend was found for serum Hp concentrations, which started to rise after 24 hours and peaked at 60–68 hours after both challenges, reaching, on average, 1.70 g/l (± 0.68) in the first challenge and 1.13 g/l (± 0.08) in the second challenge. Haptoglobin concentrations in serum then decreased and were on average 0.61 g/l (± 0.54) by 6 days PC after the first challenge and 0.23 g/l (± 0.10) after the second challenge. Serum Hp concentrations were significantly lower in the later challenge (*p *< 0.001; Figure 5)

The basic concentrations of serum LBP before the challenges were on average 10.8 mg/l (± 7.7) after the first challenge and 10.0 mg/l (± 6.4) after the second. Serum LBP started to increase rapidly in both groups and peaked at 36 hours PC, being on average 148.6 mg/l (± 41.8) after the first challenge and 108.9 mg/l (± 31.6) after the second. No statistically significant difference was recorded between the challenges (Figure 5).

WBC started to decrease after both challenges, being at the lowest 12 h PC (on average 2.03 × 10^9 ^cells/l at first and 2.97 × 10^9 ^cells/l at second challenge), then starting to increase, being at its highest an average of 8.61 × 10^9 ^cells/l (range 4.85–10.9 × 10^9 ^cells/l) at 60 hours after the first challenge and at 24 hours 10.47 × 10^9 ^cells/l (8.02–15.8 × 10^9 ^cells/l) after the second. WBC levels were higher after the second challenge during the whole experiment. The difference in WBC levels was statistically significant (*p *< 0.05; Figure 5).

## Discussion

Using a repeated challenge model at a short interval in the same cows could reveal possible carry-over effects of the previous intramammary infection by the same pathogen [22]. In our study using two consecutive intramammary challenges with *E. coli*, all cows became infected and developed local (swelling, soreness, clots in milk) and systemic inflammatory reaction. Cows had a moderate systemic clinical response to both challenges, but after the second challenge local signs were significantly milder and disappeared faster. The same pattern was seen for the indicators of inflammation, the difference being statistically significant for serum and milk Hp, milk SAA, and WBC. Milk production returned to the pre-challenge level significantly faster after the second challenge. In the present study, one dose of anti-inflammatory medication was used at 12 h PC which may slightly affect the inflammatory response but given at both challenges, allows comparison of the two subsequent challenges.

In previous studies using an experimentally induced *E. coli *mastitis model and a 3 week interval, the disease was slightly milder after the second challenge, but the differences were not statistically significant [29,27,28]. Repeated challenges with LPS at 24 h intervals were studied by Rainard & Paape [38], and observed sensitization of the mammary gland followed the first contact with a moderate dose of LPS. They did not find systemic signs after the first LPS challenge, which was speculated to be due to too small an amount of LPS to trigger the systemic inflammation response, but after the second infusion 24 hours later the systemic signs were observed. We used relatively large numbers of live *E. coli *in our challenges with a much longer interval, which resulted in a rapid inflammatory response with systemic and local signs in both challenges.

Recognition of LPS is an important event in the activation of the innate immune response to Gram-negative bacteria. LPS directly interacts with neutrophils through CD14 that is expressed on cell surfaces [39]. The effective elimination of the bacteria by neutrophils is important for the resolution of infection. If delayed, the disease can lead to development of toxemia and septic shock [3]. Some immunization effect could have occurred and resulted in a faster response and milder disease (Figure 1), as well as faster elimination of bacteria from the infected gland after prior infection in a different quarter (Figure 2). Smith *et al. *(1999) [40] showed that subcutaneous plus intramammary immunization with *E. coli *J5 bacterin produced enhanced antibody titers in milk and serum, but this not reduce clinical signs following challenge with *E. coli*. One hypothesis for the potential mechanism of action of *E. coli *vaccine is an enhanced PMN diapedesis caused by mammary gland hyper-responsiveness [41]. Recently it was suggested that the positive effect of vaccination is associated with a memory antibody response of IgG1 and IgG2 isotypes [8]. The immunological mechanism for the immunization effect seen in the present study remains unknown.

Only few studies have reported concentrations of acute phase proteins in the milk during experimentally induced *E. coli *mastitis. In the study by Jacobsen *et al. *[23], with a lower dose (50 cfu) of *E. coli*, concentrations of SAA in plasma were at a similar level, but those in the milk were 5-times as high as found here. In that study milk concentrations of SAA were highest in cows with severe mastitis but did not differ between those with moderate or mild signs. Concentrations of mammary-derived SAA in milk were many times higher than concentrations of systemic SAA in serum in their study and in ours. SAA has been suggested to have an important role in the modulation of the host response during infection [42,43]. It has been shown to bind outer membrane protein A of *E. coli*, which may also contribute to recognition of Gram-negative bacteria of the host [44]. Rapid mammary SAA response is probably involved in the innate local protection against pathogens invading the udder.

The concentrations of Hp found in the milk were similar to those reported in our previous study on *E. coli *mastitis [34]. In a study using LPS challenge [15], the concentrations of Hp increased by the end of the 12 h follow-up period and were less than half of the concentrations seen here. In the present and in the cited study where an ELISA assay was used [15], the concentrations of Hp found in milk were approximately half of those in serum. Hp assay used here has not been validated for milk, thus the results should be interpreted with some caution. The local production of Hp seems not to be so pronounced as that of SAA. Hp binds harmful molecules produced after tissue damage, such as haemoglobin, which then becomes inaccessible for bacteria by limiting their growth [45]. Hp may play a role in host defense against *E. coli *mastitis.

Concentrations of LBP in milk and plasma have been shown to increase after intramammary challenge with LPS [11] and *E. coli *[12,46]. Concentrations of LBP in blood and milk found here are higher than reported in previous studies using *E. coli *challenge models. In our study, concentrations in the milk were higher than those in blood, contrary to the findings by Bannerman et al. [12]. Challenge models and other methods may be different, which may partly explain differences between results from different studies. LBP is a hepatocyte-derived protein that binds LPS, facilitating the transfer of LPS to membrane-associated CD14 present on cells of monocytic lineage and neutrophils [47]. It enhances LPS-CD14-complex formation and thus increases the sensitivity of the host innate response to Gram-negative bacteria [47-49], having an important role in the defense of the mammary gland. It is possible that LBP is also produced locally by the mammary epithelial cells, as also suggested by Bannerman *et al. *[11], which would explain the high concentrations seen in milk.

## Conclusion

The concentrations of SAA and Hp in serum and milk in this *E. coli *infection model were much higher than those seen in experiments using Gram-positive pathogens, which indicates the strong inflammation induced by *E. coli *[22,19,50]. Acute phase proteins studied here have been suggested as early markers of mastitis. They would also be useful parameters to monitor the severity of mastitis, to be used, for example, in studies on pathogenesis and effects of treatments. Repeated experimental intramammary induction of the same animals with *E. coli *bacteria has been used as a model in cross-over studies to reduce the individual variation between different cows. The significant differences between the consecutive challenges seen here suggest that in these studies the interval between challenges should be longer than 2 weeks.

## Authors' contributions

LS was involved in the conception of the study, carried out the experiments, interpretated the results, drafted the manuscript and carried out coordination among authors, TO carried out laboratory analyses of acute phase proteins, statistical analysis and interpretation of the results and drafted the manuscript, HJ and JS carried out the experiments and participated in drafting the manuscript, SP made substantial contribution to conception of the study and revised the manuscript for important intellectual content in detail.
